# Ovariotomy

**Published:** 1875-06

**Authors:** 


### OVARIOTOMY.

By A. COUVERT, M.D.

Messrs. Editors: You know I am loth to appear in print, and shrink from notoriety. Nevertheless, I must give you a brief statement, for what it is worth. I have performed ovariotomy, *secundum artem*, very many, perhaps a thousand times, and with marvelous success. Dr. McDowell, and his worthy and illustrious successors, (you among them) would lose lustre before me, but for one unfortunate *little* fact, namely: my patients, although females, were not women; not one, but all swine, every hoof of them. Not even one biped to swear by, stir my ambition, or elevate me from my deep retirement and profound obscurity. Well, what of all that? you ask. Several things, I answer. Dr. McD. lived and practiced in a country where swine abounded, hogs being the main staple of his rural patrons. Therefore, much spaying was necessarily done. He, doubtless, witnessed the operation, and, as a pioneer surgeon, operated himself sometimes, *con amore*, and saw the harmless results. This aroused his inquiring mind; whereas, the city surgeon, of equal worth, who never sees hog but when brought before him at the *table d'hote*, in the form of ham, might never acquire a hint in human ovariotomy from such operation on inferior animals. It is not considered by any means disreputable or degrading to professional dignity to practice vivisection, or test medicines on cats and dogs, rabbits and frogs. Now, with all humility and deference, I claim for the porcine grunters equal respectability to the aforesaid subjects of the chirurgeon's knife, and ask, why not accept the teachings of these experiments, already made, (if indeed they teach anything) though made for a different purpose. I am no surgeon, have devoted no special attention to that branch of the profession; but before the war I owned and lived on a large farm, and for years spayed many of my hogs of all sizes, until I taught an expert servant to relieve me of it, as it often conflicted with important duties, more in harmony with my taste. But to the point. Dr. McD., I assume, saw how little the operation hurt the hog; how few die, or even miss a feed, when spayed in the side; how effused blood, sometimes in large quantities, falling into the abdomen, (both from the parietal incision and the vessels of the excised ovaries,) does no harm, though never discharged; this being prevented by close sutures and the unvarying fact that the hog lies with the cut side up until the wound is healed. There is another mode of spaying, to-wit: in the median line. This is easier to the operator, but worse for the hog, who droops for days, and many die. From a hog thus operated on the blood may be discharged by its own gravity, if not prevented by tight sutures; but such discharge never takes place, in either mode, except in such cases as terminate fatally.

Some of the points that seem well established by experience are: 1st. Effused blood into the cavity of the abdomen does no harm—to a hog. 2d. The hog, when cut through the thick muscles of the side, suffers less and recovers sooner than when cut through the thinner but tendonous median line. Many die when thus cut; rarely one dies when cut in the side. This may be due, in part, to the tissues involved, and partly to the weight of the viscera, straining the sutures in one case, thus opening the wound; and in the other, keeping the lips in apposition, almost without the need of a stitch. Usually, the incision in the median line requires three sutures; the side but one.

But I have written enough to evoke whatever there may be in the subject worthy of consideration, by you or others, so well versed in human ovariotomy, if, indeed, there is any relevancy of one to the other.

